# Welcome, Orientation, Language Training: a project at the Charité for new international medical students

**DOI:** 10.3205/zma001205

**Published:** 2018-11-30

**Authors:** Wendelin Marmon, Ulrike Arnold, Asja Maaz, Marwa Schumann, Harm Peters

**Affiliations:** 1 Charité – Universitätsmedizin Berlin, Charité International Cooperation (ChiC), Berlin, Germany; 2 Charité – Universitätsmedizin Berlin, Dieter Scheffner Fachzentrum für Medizinische Hochschullehre und evidenzbasierte Ausbildungsforschung, Prodekanat für Studium und Lehre, Berlin, Germany

**Keywords:** international students, undergraduate medical education, preparation courses

## Abstract

**Objective: **A comprehensive, integrated support programme for new international students of medicine has been developed, implemented and evaluated at the Charité. The objectives of the programme were improved social integration, orientation on the study program and Charité campus, as well as qualification in medical specialist language.

**Project outline: **The “Charité Orientation Module for International Students” (ChOIS) was designed by a working group with a variety of expertise in the field of international students. The programme has three stages:

1. Recruitment (specific invitation on matriculation);
2. Orientation week before semester start; and
3. Parallel events during the first semester.

ChoOIS was piloted in the Winter Semester 2015/16 and, following evaluation, continued in a modified form in the Summer Semester 2016. Key features were:

1. Welcome and social integration by faculty welcome-events and student group activities;
2. Orientation on the study program, on teaching infrastructures at the Charité and on student life in Berlin by senior medical students; and
3. Training in language for medical communication and bedside teaching by professional lecturers.

**Results:** Results of evaluations conducted after the orientation weeks, at the end of the semester and retrospectively in the 3rd semester produced high approval ratings of the individual features of the ChOIS-programme and of the programme as a whole by participating students.

**Discussion: **A comprehensive, integrated support programme for new international students of medicine has been developed and implemented. The ChOIS-programme can serve as a practice model to guide other medical faculties. In future, a programme that goes beyond the start of the course and includes more involvement by senior students would be desirable.

## Zusammenfassung

**Zielsetzung: **Für internationale Medizinstudierende wurde an der Charité Berlin ein umfassendes, integriertes Unterstützungsprogramm für den Studienbeginn entwickelt, implementiert und evaluiert. Programmziele waren Verbesserung von sozialer Integration, Orientierung im Studium und am Studienort sowie medizinspezifische, sprachliche Qualifizierung.

**Projektbeschreibung: **Das „Charité Orientierungsmodul für Internationale Studierende“ (ChOIS) wurde von einer Arbeitsgruppe mit verschiedenen Expertisen zum Thema internationale Studierende als dreistufiges Programm konzipiert:

1. Rekrutierung (gezielte Einladung bei der Immatrikulation);
2. Orientierungswoche vor Semesterbeginn; und
3. Begleitende Veranstaltungen während des 1. Semesters.

ChOIS wurde im Wintersemester 2015/16 pilotiert und auf Basis der Evaluation im Sommeresemster 2016 modifiziert weitergeführt. Inhaltliche Schwerpunkte bildeten:

1. Willkommen heißen und soziale Integration in Veranstaltungen der Fakultät Charité und gemeinsamen Aktivitäten der Studierenden;
2. Orientierung in Studium, Charité-Lerninfrastruktur studentischem Leben in Berlin in Tutorien von Medizinstudierenden höherer Fachsemester; und
3. Sprachtraining für medizinische Kommunikation durch professionelle Dozenten für medizinische Fachsprache.

**Ergebnisse: **Die Evaluationen nach den Orientierungswochen, zum Ende des Semesters und rückblickend aus dem 3. Semester ergaben eine große Zustimmung zu den einzelnen Elementen des ChOIS-Programms und dem Programm insgesamt seitens der teilnehmenden Studierenden.

**Diskussion: **Es wurde erfolgreich ein umfassendes, integriertes Unterstützungsprogramm für internationale Medizinstudierende zum Studienstart konzipiert und implementiert. Das ChIOS-Programm kann anderen Medizinischen Fakultäten als Praxismodell der Orientierung dienen. Für die Zukunft sind ein über den Studienbeginn hinausgehendes Programm und die stärkere Einbeziehung von internationalen Studierenden aus höheren Semestern wünschenswert.

## 1. Introduction

The transition from school to a medical degree is for many, if not all, new students a significant and critical challenge on the way to becoming a doctor. Transition is defined as a “phase of change, in which people” (here medical students) “experience a certain discontinuity in their professional life which forces them to react by developing new behaviours, or to change their professional environment in order to cope with the new situation” [1]. To help with this transition, in this case the beginning of a medical degree with its relatively high expectations, many faculties offer specific support programmes according to the needs of the majority or larger group of students.

International students, who have completed their education so far in another country, and who travel to a country to study (foreign students), form a significant group. For these students, the “normal” burden of transition is increased due to e.g. barriers in medical communication and social integration [2], [3], and they have additional specific needs [4], [5]. These are rooted in the expectation of medical communication with lecturers or fellow students, with patients or in teams of doctors or interprofessional groups [3], [4]. A particularly challenging transition for this group is that of German changing from a foreign language to one used every day. Added to this, there is a specific need for social integration in and outside of the course. Empirical studies show that international students perform worse in written and oral examinations and take longer to complete their studies [2], [6], [7], [8], [9], [10], [11].

More than 2000 international students enrol at medical faculties in Germany each year [4]. This is around 15% of all medical students [4]. The number of international students has increased continually in recent decades [12]. This trend is very likely to continue in the future. According to a recent review, support services for this group vary and are mostly sporadic [4]. These include student-led tutorials, “German for Medics” language courses, partnership schemes of senior students with new international students, specific counselling services, examination preparation courses or group leisure activities. At many German medical faculties, however, the current situation is widely regarded to be unsatisfactory [4]. Communication problems in the medical setting and the social integration of international students are at the forefront of the issue. International students themselves have expressed an added need for support with financial problems as well as intercultural exchange [5]. Apart from a peer-assisted learning program for exam preparation [12], there is a lack of systematically developed and evaluated practical examples of effective support programs for international medical students at German medical faculties. Published literature on this subject in German speaking countries is sparse, and in particular, it lacks practical examples.

In this project outline, we present an integrated support programme developed at the Charité – Universitätsmedizin Berlin (Charité) for new international students as a guide for other faculties who may be developing or improving their own programmes. The systematic development and implementation of the programme “Charité Orientierungsmodul für Internationale Studierende” (Translated: Charité Orientation Module for International Students, hereafter ChOIS) was based on the experience of an interdisciplinary, curricular design team as well as current literature. We describe the structure, scope and content of the ChOIS-Programme and its efficacy based on student evaluation.

## 2. Project Outline

### 2.1. Concept

The concept for ChOIS was designed over three months by an interdisciplinary team: the director or the Charité International Cooperation (ChiC), a German teacher specialising in German as a foreign language for medical communication, and a medical student in the 5^th^ year of study. The team’s own experiences, together with published literature, formed the basis of their planning. In six planning meetings, three key aspects were first established (social integration, orientation support and linguistic qualification in medical communication), followed by detailed planning in consensual teamwork. 

#### 2.2. Process and structure

The ChOIS-programme is structured in three phases: recruitment, orientation week prior to the start of the course, and sessions throughout the first semester.

**Recruitment**. Information leaflets were on display in the admissions office (Referat für Studienangelegenheiten) for international students on matriculation. Those interested were invited to register for the programme via email to ChIC. Reflecting a ratio of 10% EU-citizens and 5% other per cohort/ course-start, around 30-35 international students were eligible for the programme.

**ChOIS-Programme structure and procedure**. An overview of the structure, elements and content of the ChOIS-Programme can be found in Table 1 (Tab. 1), which shows the orientation week before the beginning of the study program and Table 2 (Tab. 2), which shows the events accompanying the first semester. The three key components were organized as follows:

##### Welcome and social integration

At the beginning of orientation week, international students were officially welcomed by the Dean of Student Affairs and ChiC-staff. During orientation week, in an intensive, one-day small-group session, we encouraged students to get to know each other and to see that they were not alone with their non-German typical problems. Pair work and small group activities as well as open discussions in plenary-like sessions contributed to team-building, while at the same time preparing students for the teaching formats on the modular degree course.

Several events throughout the semester further aimed to improve social integration. An “international dinner” for instance, for which each guest provided a speciality from their country, facilitated private exchange in a relaxed setting away from studies and exams. On the second and third runs of these events, senior international students were also invited. 

The programme concluded with a celebratory event in collaboration with the Dean of Student Affairs. Participating students received a certificate, symbolizing that, as part of a welcome culture at a higher institutional level, international students are taken notice of and respected. 

##### Orientation on the study program, on teaching infrastructures at the Charité, and on student life in Berlin

The first semester of the modular degree in medicine begins with an introduction week for all students. Large events in quick succession feed students with an almost overwhelming amount of new information with new terminology, which is a challenge even for native speakers. We decided explicitly to deal with a number of topics in an orientation week for international students before introduction week. Sessions dealt with aspects of the course that were more difficult to understand in detail at first sight. These included an introduction to the unique features of the modular degree and its specific teaching formats, an overview of first semester content, a presentation of possible learning strategies and an introduction to the university online platforms, the skills lab and student tutorials. Important information for student life (accommodation, public transport, cycling, extra activities in and outside of the university, life-study balance), a tour of the campus and a walk through the city were also offered.

In the accompanying sessions during the first semester, students were given general information and tips for exam preparation towards the end of the semester. Our student tutor explained the multiple-choice format used at the Charité and based on her own experience, presented learning strategies for effective preparation. As well as this, they received information on the obligatory nursing internship, which most students start at the end of the first semester. These sessions also provided international students with the space to ask questions and discuss any problems. In addition, regular emails were sent with reminders and offers of support with tasks such as registering for exams, finding internships etc.. 

##### Training in language for medical communication and bedside teaching

In determining the need for language support, we looked at the core content of the first semester, concentrating on specific language and communication aspects. Through this, we determined what study material would be particularly challenging to non-German students at a linguistic level and where support would make sense. In early clinical teaching in the modular curriculum of medicine at the Charité these emerged mainly as “Basic knowledge of the human body” and “Taking a patient’s medical history and physical examination”.

The subject “Basic knowledge of the human body” was mainly dealt with in the ChOIS orientation week at the beginning of the semester. The aim was that students would be able to use basic knowledge about the human body that would be expected from any newly enrolled student in the German language. This took away some of the burden of acquiring new knowledge in a foreign language later. A central learning objective was to create an awareness that doctors move on several linguistic levels (specialist terminology vs. colloquial language, transfer language) [13]. A further aim was to prepare students for basic fields of human medicine, e.g. anatomical terms, organ and metabolic functions, symptoms and physical sensations.

Doctor-patient communication while taking the patient’s medical history and carrying out an examination is, from a language perspective, one of the most demanding tasks for doctors [14]. In this situation, words and expressions are used which are new to a learner of the language. The discerning language used for describing symptoms, pain and physical sensations during medical history taking, giving instructions to patients in a physical examination, while maintaining a polite, discrete, trustworthy and professional atmosphere is also achieved through language and is culturally specific. This requires a high degree of intercultural learning from international medics. The design of the training was based on our experience from communication training carried out for international doctors at the Charité international academy. The extensive training needed by non-German doctors in this field corresponded to that of new international students. This subject was mainly dealt with in sessions during the semester, after students had had some initial experience in their physical examination course. 

An overview of the orientation week can be found on Table 1 (Tab. 1).

#### 2.3. Selection of lecturers

Language teaching and cultural training were carried out by professional lecturers for German as a foreign language with experience in teaching medical German at universities. Orientation seminars were carried out by medical students in higher semesters.

#### 2.4. Evaluation

The ChOIS programme was evaluated at the end of the orientation week, at the end of the programme (end of first semester) and retrospectively in the third semester.

## 3. Results

### 3.1. Implementation

The ChOIS-programme was piloted in the Winter Semester of 2015/2016 and, as described in this article, ran a second time in the Summer Semester of 2016. Following evaluation, the following modifications were made: the social integration aspect was expanded with more group activities and involvement of higher semester international students. There was less emphasis on culture, history and orientation in Berlin. The language aspects were felt by some to be too simple. During planning, we had not taken into consideration that there were some fluent German speakers among the international participants. To cater to this heterogeneity, language training was split into sessions for those with especially high needs and sessions for all participants.

#### 3.2. Participants

The origin of the international students (n=22) was as follows: Europe: 2 students from Russia, 1 each from Bosnia and Herzegovina, Bulgaria, Greece, UK, Montenegro and Poland. Near and Middle East: 6 students from Syria, 1 each from Armenia and Saudi Arabia. Asia: 1 each from India and South Korea. Africa: one student from Cameroon. North America: 2 students from the USA. The most common first languages were Arabic and English.

#### 3.3. Evaluation

Table 1 (Tab. 1) (orientation week) and Table 2 (Tab. 2) (accompanying seminars in the first semester) give an overview of the results of the quantitative evaluation. 

The evaluations show that approval for our service improved significantly the second time round. In the course orientation tutorials, the sessions on the content of the first semester and the use of online platforms were rated best. Among the language training modules, “Communication on the ward/for nursing” and “Taking a patient’s medical history and physical examination” were rated as particularly good. Several participants expressed a desire for more of this content. 

On the whole the following aspects were rated as especially helpful: “social (meeting people etc.)” at 100%, “orientation on the course and organising my studies” at 92% and the language aspects at 88%.

#### 3.4. Scope and necessary resources

The orientation week had a scope of approx. 45 teaching units of 45 minutes (including informal/evening events). The parallel sessions comprised 9 evening seminars with 20 teaching units. Lecturers carried out 35 teaching units while student tutors led 38. 

A detailed project description including detailed evaluation results and best practice examples for lesson planning can be found as a Charité International Cooperation online brochure [15]. 

## 4. Discussion

The transition from school to the medical degree is not only a particular challenge for international students, but also for hosting faculties. Although sporadic support services are established at many medical faculties in Germany [4], the ChOIS-programme, based on international recommendations [16], the planning and successful implementation of a comprehensive, integrated programme at the start of the course has been a success. The three key aspects “Welcome, orientation, language training” will be discussed in the following.

The aspects of the ChOIS-programme for strengthening social integration received universally positive evaluations. The lowering of language barriers [17] and with it, the opening of possibilities to participate more in the social side of student life, played a central part in this. This provided a basis for more intensive cultural exchange and potentially for higher performance levels [18]. In our view, the orientation support offered was also an essential part of the programme, as it enabled international students to have a better start to their course, with important information being conveyed to the right target group. 

The language training offered by the ChOIS-programme enabled students to improve their communication skills particularly with patients and lecturers. These language courses are gaining significance, due especially to the often debated language assessments as part of admission to university, which focus on general language skills [4]. An improvement in communication skills enables international students to provide better care for patients [2]. It should be stressed that these language courses were held by a professional lecturer. 

The ChOIS-program only supports international students at the beginning of their course. A program which supports students throughout their course would surely also be desirable. This could support students at further transitional points, such as the first clinical clerkship, taking the state examinations or at the start of the practical year [16]. For the future development of the ChOIS-programme, international students from higher semesters should be involved as central stakeholders, as part of student engagement measures, both in its design and as personnel. International students should also be more in contact with their fellow students, achievable with e.g. a buddy programme, whereby a German fellow student becomes a mentor (buddy). In view of the increasing number of international medical students, future research activities [19] should focus on highlighting the needs of and disadvantages for international students, creating support schemes, and gaining deeper knowledge of progress, success and drop-out rates, so that the gap in services for support and integration at medical faculties can be closed. 

The ChOIS-programme has limitations. It has been implemented at one medical faculty and has thus been tailored to the local situation e.g. the modular curriculum of medicine or early clinical teaching. The evaluation took place mainly through quantitative methods and concentrated on level 1 of Kirkpatrick’s model for training course evaluation [20]. Effects on learning success or the acquisition of medical competencies and their application during clinical work were not evaluated. 

## 5. Conclusions

The ChOIS-programme has enabled us to implement a systematically designed, extensive, integrated support programme for international students at the beginning of their course. Evaluation results by participants underline the need for such a programme and its specific features. This project outline aims to provide other medical faculties with a practice example that may guide the further development of their support programmes for international students. In future, increased efforts, such as those demanded by the Stifterverband 2015 [21] e.g. additional scholarship and support programmes, need to be put into practice, so that international students are successful in their studies, socially and professionally integrated, while at the same time preventing drop-outs. 

## Funding

The ChOIS-programme was funded by the Berlin Senate Department for Integration, Work and Social Affairs as part of the funding programme “Integration through qualification”.

## Competing interests

The authors declare that they have no competing interests. 

## Figures and Tables

**Table 1:**
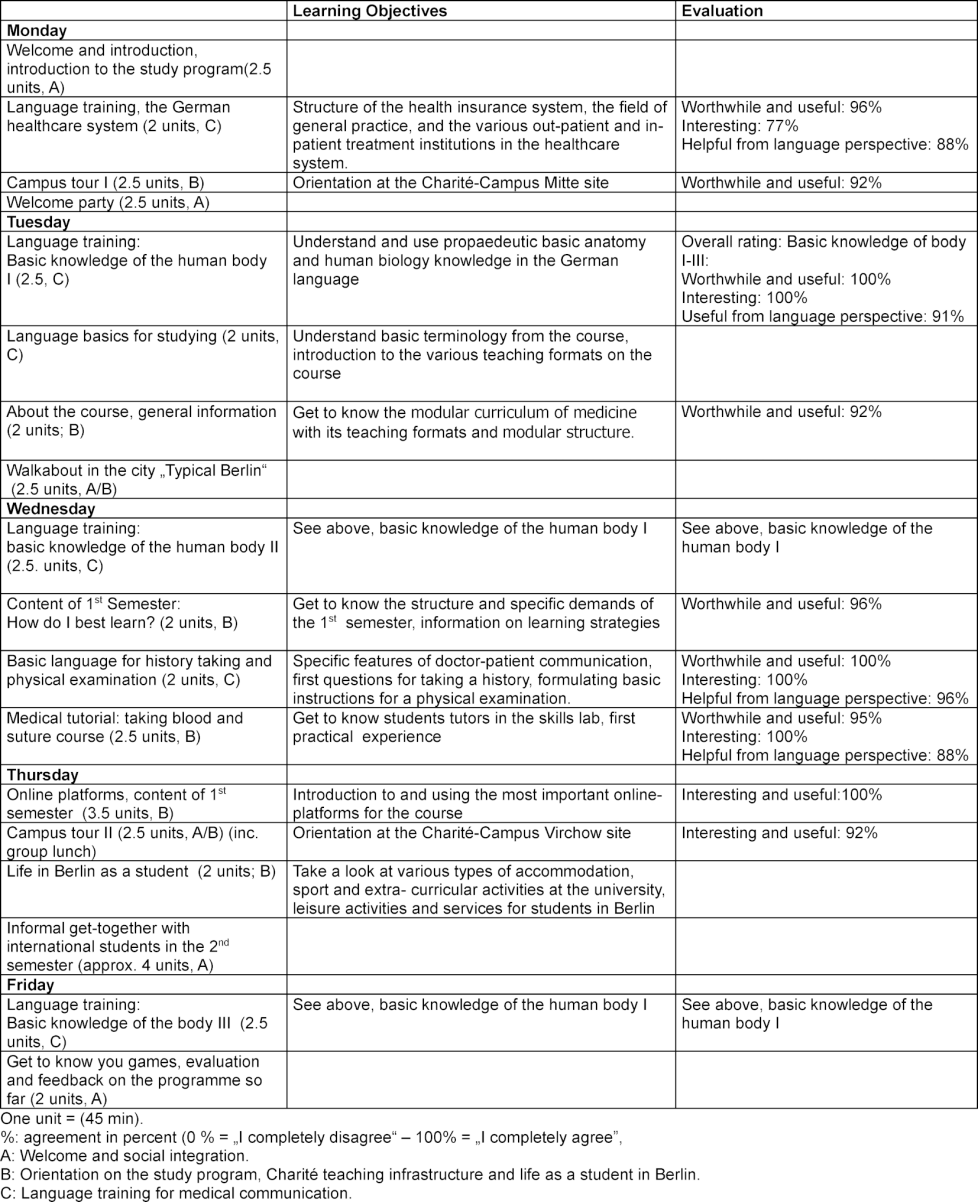
Overview of content and themes in the orientation week for international students before the study program starts

**Table 2:**
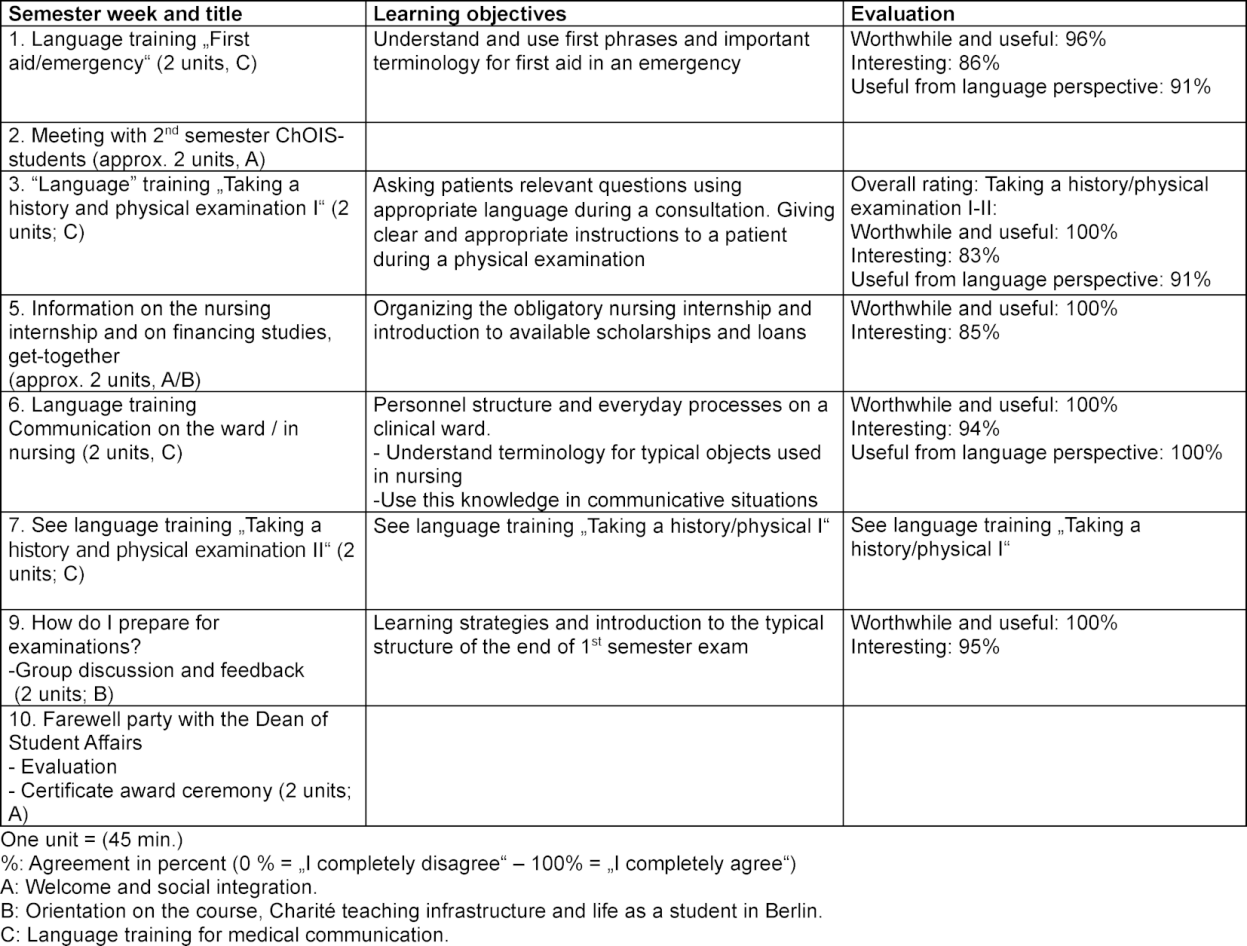
Overview of content and themes of sessions for international students during the 1^st^ semester
